# Supplementary material for: Are carbon emissions trading and green financial instruments synergistic? -Comprehensive quantitative research based on content analysis
Source: PLoS One. 2024 Mar 7;19(3):e0298601. doi: 10.1371/journal.pone.0298601 (PMC10919601; doi:10.1371/journal.pone.0298601)
Supplement: S1 Data — (ZIP) [file pone.0298601.s001.zip › Data/地级市Z_F工作报告.zip!/╣π╢1⁄2/╓╨╔╜╩╨/└┤╫╘╣1⁄2╓┌║┼í╛┬φ┐╦╩2╛▌═°í┐_2012.docx]

过去五年工作回顾

　　本届政府任期五年，是我市成功应对各种严峻考验，取得科学发展显著成就的五年。市政府在省委、省政府和市委坚强领导下，在市人大及其常委会和市政协监督支持下，在全市人民共同努力下，以邓小平理论和“三个代表”重要思想为指导，深入贯彻落实科学发展观，大力推进经济强市、和谐中山、宜居城市、法治社会、文化名城和民生工程建设，胜利完成市十三届人大一次会议确定的目标任务，实现经济社会发展新跨越。

　　这五年，是综合实力显著增强的五年。预计全市生产总值跨越2000亿元大关；人均生产总值从4.2万元增加到7.1万元，在常住人口增长22.5%情况下增长65%；工业总产值增加到6200亿元。生产总值、工业总产值、财政一般预算收入、全社会固定资产投资、社会消费品零售总额均翻番增长，经济实力保持全省第5位。产业高级化明显，三次产业结构调整为2.7:55.8:41.5，高新技术产品产值年均增长20%，装备制造业产值年均增长19.3%。规模以上工业企业利润总额增长1.6倍，服务业增加值占生产总值比重提高5.4个百分点。产值超亿元企业从480家增加到759家，拥有上市公司18家。实际利用外资38亿美元，出口总值增长61%，保税物流中心投入运营。共建4个省级产业转移园区。科技创新能力增强，发明专利授权量增长10倍，新增省级以上工程中心和技术中心46家，连续五届获评全国科技进步考核先进市。主要污染物总量减排“十一五”考核全省第一。

　　这五年，是城市建设显著变化的五年。组织投入248亿元发展交通，构建“五横六纵七高速九加密”路网。广珠西线二期、西部沿海高速公路珠海段支线、古神公路一期、城桂路二期、福源路三角段、番中公路民众跨线桥立交等建成通车，公路密度从每百平方公里92.5公里提高到109公里。实现轨道交通零突破，6个城轨站投入使用。“一港五区”建设完善，港口进出口货物吞吐量累计560万标箱。嘉明电厂二期等一批能源项目投入使用。水利防灾减灾工程全面完成省下达任务。建成20个污水处理厂和669公里管网，城镇生活污水集中处理率87.5%。中心组团、北部组团垃圾综合处理基地投产运营，城区生活垃圾无害化处理率100%。构建“一核一环多廊多点”绿地体系，省立绿道中山段全线贯通。建成16个国家生态示范镇，成为国家生态市。

　　这五年，是统筹城乡显著提升的五年。城镇化率达87.9%。城镇居民人均可支配收入超过2.7万元，农村居民人均纯收入超过1.7万元，年均增长7%和12%，城乡居民收入差距全省最小。市财政投入支农资金78亿元，农村基础设施日趋完善，城乡公路公交、宽带网络等全覆盖。建设规模化农业生产基地43个、现代农业园区8万亩，农业亩产值增长73%。建设59个农民集中居住小区，完成779公里主干农路“硬底化”。农村综合改革稳步进行，农村土地流转率95%，完成农村股份合作制和村级统一核算改革。

　　这五年，是体制改革显著推进的五年。创新行政审批制度，推行“一网式”审批，开设电子监察系统。新一轮政府机构改革基本完成，事业单位人事制度改革取得阶段性成果，开展简政强镇事权改革。推行政府投资项目代建制，鼓励民间投资。构建公共财政管理新机制，对欠发达镇实施“一级财政”、“专项补助”政策。率先在全国实施流动人口积分服务管理。落实《珠三角规划纲要》，加强珠中江合作，牵头实现机动车年票互认，积极参与粤港澳台区域合作。

　　这五年，是社会民生显著改善的五年。财政投入社会保险13.7亿元，率先建立城乡一体社保体系，实现城乡社会基本养老保险、住院和门诊基本医疗保险全覆盖，城镇职工社保总参保人次由371.2万增加到705.6万，城镇养老保险待遇由人均每月924元提高到1736元，基本医疗保险报销额最高达30万元。建立城乡一体全覆盖就业制度，创建国家级创业型城市，农村劳动力转移就业考评全省第一。社会救助救济体系不断完善，慈善万人行获中华慈善奖。市财政投入158亿元优先发展教育，所有镇区成为省教育强镇，实现免费义务教育，高中阶段毛入学率100%，高等教育普及化，职业教育超常规发展。医疗卫生服务水平不断提升，建成国家级卫生镇11个，所有村（社区）建有卫生站。实施孙中山文化等八大文化工程，成为国家历史文化名城。公共文化设施覆盖城乡，所有镇区文化站达省特级标准。获评全国计划生育优质服务先进单位，户籍人口年均自然增长4.8‰。全民健身运动广泛开展，竞技体育成绩显著。蝉联全国双拥模范城。圆满完成漩口援建任务，“双到”扶贫工作走在全省前列。安全生产和平安建设持续向好，交通、火灾事故逐年下降，连续四届获评全国社会治安综合治理优秀地市。全国文明城市建设成效扎实。

　　这五年，是行政服务显著增效的五年。坚持依法行政，自觉接受市人大依法监督和市政协民主监督。坚持重大工作向市委、市人大报告和向市政协通报制度，五年办理人大代表议案建议541件，政协提案1071件，答复率为100%。率先建立行政复议委员会和镇区行政复议受理点，规范行政处罚自由裁量权经验在全省推广。加快政府职能转变，开展政务公开和网络问政，拓宽民意表达渠道。强化信访机制创新，构建“大调解”格局，设立人民调解委员会537个、基层劳动争议调解委员会481个、综治信访维稳工作站279个，扎实开展普法和法律援助工作，社会大局和谐稳定。

　　刚刚过去的2011年，是本届政府任期最后一年。市政府紧紧把握纪念辛亥革命一百周年重要际遇，围绕率先加快转型升级、建设幸福和美中山的核心任务，以抓转型、促发展、优布局、强交通、惠民生为重点，一心一意谋发展，聚精会神搞建设，全力以赴抓落实，经济社会发展全面迈上新台阶。

　　经济平稳较快增长，“十二五”开局良好。预计全市生产总值2182亿元，增长13.5%，人均生产总值增长12.5%；工业增加值1154.1亿元，增长14.3%；服务业增加值905.5亿元，增长13%；财政一般预算收入183.1亿元，增长31.3%；全社会固定资产投资754.6亿元，增长20%；出口总值251亿美元，增长11.5%；社会消费品零售总额758亿元，增长16.9%。

　　转型升级步伐加快，发展质量不断提升。“三个一百”等系列政策显现成效。引进一百首批50个项目总投资780亿元，土地投资强度和产出效益明显提高。内资百强企业销售收入和税收增长均超20%。百家外资企业及来料加工企业转型升级进展良好，加工贸易内销增长8%，提前完成省下达任务。5145家个体工商户转型升级为企业。“3•28”和央企、民企、世界500强招商引资总额创历史新高。研发投入占生产总值比重2.1%，国家知识产权快速维权中心落户我市，引进3家国家级创新平台，火炬区成为国家创新型科技园区。重奖科技创新人才，实现国家和省专利金奖零突破。积极推进产业和劳动力“双转移”。实施“三旧”改造3万亩，获得省节约集约用地一等奖。规模以上工业全员劳动生产率提高17%，经济综合效益指数提高10个百分点。

　　基础建设加大力度，城市面貌明显改善。翠亨新区列入省“十二五”规划预备项目，翠亨国际旅游小镇进入规划实施阶段。主城区建设扩至“四区六镇”，城市管理覆盖城乡。大力推进交通畅行，新岐江公路、新十水线古镇至小榄段、阜港公路和城桂路改造等建成通车，广珠中线二期、广珠西线三期、东部快线、小榄快线、福源路三角至港口段等加快推进。建成智能交通二期工程，投入334辆新能源公交车，公交出行分担率达20%。建成58公里城市慢行道，投放4000辆公共自行车，绿色出行成为新风尚。新种树木30万株，城市绿化档次明显提升。全禄蓄淡抗咸工程、长江水厂扩建等项目竣工使用，岐江游愿景成为现实。

　　环境保护加速推进，生态文明取得突破。通过国家环保模范城市复检，建设全国生态文明示范市。前山河流域、内河涌和岐江河治理力度加大，珠江综合整治考核居全省前列，获评“八年江水变清”优秀城市。南部组团垃圾综合处理基地、固体废物处理中心建设加快。完成林分改造7500亩，森林覆盖率19.3%。单位生产总值能耗下降4.1%，全年空气优良率99.8%。

　　民生保障持续改善，社会事业和谐共进。市财政重点民生支出136.1亿元，增长38.8%。新增就业岗位5.1万个，城镇登记失业率控制在2.1%以下。建设71家平价商店、蔬菜基地和冷库，居民消费价格涨幅控制在5%左右。全民修身行动成为全省试点。数字电视整体转换19万户，“农村文化室全覆盖”成为全国公共文化服务示范。落实传染病防控等基本公共卫生项目，国家卫生城市通过复审，食品药品安全进一步加强。筹建保障性住房8609套，超额完成省下达任务。实行低保标准城乡一体化，完成低保家庭危房改造572套，为2万户老人安装爱心铃。率先实现镇区社工服务中心全覆盖，获评中国社会管理创新城市，“2＋8＋N”模式成为全国农村社区建设实验全覆盖示范。和谐劳动关系八项机制在全国推广。社会治安视频监控系统5891个点覆盖城乡，各类生产安全事故宗数、直接经济损失分别下降2.4%和22.3%。外事侨务、港澳事务、台务工作服务发展贡献明显，民族宗教工作促进和谐成效突出，统计、审计、国防建设、妇女儿童、老龄、残疾人、气象、人防、档案、方志等工作成绩显著。

　　辛亥纪念圆满成功，伟人故里影响增强。贯彻“惠民是最好纪念、发展是最好继承”理念，成功举办四大系列百项活动。投资2000亿元42个重点项目启动和开竣工，孙中山文化节和辛亥革命纪念公园、孙文公园名人雕塑、兴中园主题雕塑等一批重点项目集体亮相。实现对内凝心聚力、对外提升形象，促进转型升级、民生福祉、市民素质、城乡面貌和城市竞争力的全面提升，增强了伟人故里的凝聚力与影响力。

　　各位代表，回顾走过的历程，我们深深体会到：

　　坚持以科学发展为第一要务，是我们走向跨越的重要法宝。牢牢把握经济建设中心不动摇，脚踏实地干实业，专心致志促发展；牢牢抓住转型升级不放松，推动经济增长从要素驱动向创新驱动转变、从外源驱动向内外源协同驱动转变、从资源驱动向劳动者素质驱动转变，提高发展质量效益。

　　坚持以造福于民为目的，是我们履行使命的本质要求。把为人民谋幸福作为工作的出发点和落脚点，为人民办好事、做实事、解难事，做到发展为了人民、发展依靠人民、发展成果由人民共享；统筹推进经济、社会、文化、生态、民生建设，促进人与自然和谐相处，让人民享受可持续发展的幸福。

　　坚持以改革创新为动力，是我们奋发进取的智慧源泉。解放思想、求真务实，从中山实际出发，创造性地开展工作，不断提高政府推动科学发展、促进社会和谐的能力；善谋善治、先行先试，深化重点领域和关键环节改革，积极应对前进道路上的各种严峻考验，不断开创工作新局面。

　　坚持以和谐稳定为基础，是我们加快发展的重要保障。正确处理改革发展稳定的关系，倍加珍惜团结、安定、和谐的社会环境，倍加爱护包容、博爱、向上的良好局面；积极做好新形势下群众工作，妥善解决新时期社会矛盾，在全社会形成心齐气顺、劲足实干的优良环境。

　　坚持以共同事业为感召，是我们凝心聚力的优良传统。以建设幸福和美家园的共同事业形成共识、集聚资源，增强合力、奋勇争先；传承孙中山精神，发挥侨乡优势，汇聚海内外乡亲和新老中山人热爱家乡、建设家园的智慧力量，共同建设伟人故里。

　　各位代表，五年的经验，是我们弥足珍贵的精神财富和继续前进的行动指南；五年的奋斗，为更高水平地建设“三个适宜”幸福和美家园打下了重要基础；五年的成就，使中山站在了率先加快科学发展的崭新起点。这是市委总揽全局、正确领导的结果，是市人大和市政协监督支持的结果，是各民主党派、无党派人士和衷共济的结果，是全市人民团结奋进的结果，是海内外乡亲及社会各界鼎力相助的结果。在此，我代表市政府，向全市广大工人、农民、知识分子、企业家、干部群众，向各民主党派、工商联、各人民团体、无党派人士和社会各界人士，向驻我市解放军和武警官兵、公安民警，向所有关心支持中山发展的老领导、老同志、港澳台同胞、海外侨胞和国内外友人，表示衷心的感谢和崇高的敬意！

　　我们清醒认识到，我们的工作与人民的期待还有差距，经济社会发展还存在不少问题。突出表现为“六个矛盾”：产业结构升级迫切与粗放型发展方式的矛盾；经济快速增长与土地资源紧缺和环境承载力有限的矛盾；工业化进程加快与城市化水平相对滞后的矛盾；人口机械增长过快与高素质人才短缺的矛盾；社会转型加快与深层次问题不断凸显的矛盾；人民群众对公共服务的期望与政府供给能力有待提高的矛盾。这些都是我们必须破解的问题，我们要以对人民高度负责的精神，采取有力措施加以解决。

今后五年展望和2012年工作建议

　　今后五年，是我市科学发展的黄金期、转型升级的关键期、改革破题的攻坚期、跨越发展的机遇期。纵观国内外形势，我们既面临前所未有的机遇，也面临极其严峻的考验。世界经济格局发生深刻变化，我国经济社会发展长期向好基本态势没有改变，珠江口湾区世界级城市群建设力度加大，我市多年积累的良好基础不断强化，为加快发展创造了有利条件。与此同时，国际形势十分严峻复杂，世界经济复苏的不稳定性不确定性上升，我国经济增长下行压力与物价上涨压力并存，珠三角城市竞争加剧，我市经济增长内生动力不足，对我们加快发展提出了更高要求。我们要增强忧患意识和机遇意识，加强战略谋划，提高应对能力，积极主动作为，促进更长时期、更高水平、更好质量的发展。未来五年，我们要把思想行动统一到市第十三次党代会的决策部署上来，正确处理好发展速度与转型升级的关系、经济建设与社会建设的关系、强市与富民的关系、城市与产业的关系、发展与保护的关系、共建与共享的关系，把伟人故里建设得更加繁荣美丽、更加和谐幸福。

　　今后五年政府工作的总体要求是：深入贯彻落实科学发展观，认真贯彻市第十三次党代会精神，全面实施《珠三角规划纲要》和“十二五”规划纲要。强化发展的人均意识、集约意识、精品意识和统筹意识。着力产业升级，提升核心竞争力；着力城市转型，建设理想城市；着力民生改善，提高幸福指数；着力社会善治，促进和谐稳定；着力绿色发展，永葆碧水蓝天；着力文化引领，共建精神家园。努力率先走出具有珠江口西岸特色的科学发展道路，全面建设适宜创业、适宜创新、适宜居住的幸福和美中山。

　　未来五年政府工作的奋斗目标和主要任务是：

　　——综合实力跃上新台阶。全市生产总值年均增长11%，2016年人均生产总值超过11万元。经济发展方式转变明显，产业结构更加优化。实现速度、质量、效益相协调，消费、投资、出口相协调，人口、资源、环境相协调。城市经济实力、增长动力、创新能力显著增强。

　　——社会发展取得新成效。城乡一体社会保障体系更加完善，城镇户籍从业人员养老保险参保率接近100%，人口自然增长率不超过7‰，高等教育毛入学率超过60%，学前三年毛入园率达98%以上，国民教育体系日臻完善，人民健康水平不断提高，社会事业全面进步。

　　——城市建设呈现新面貌。城镇功能布局进一步优化，“一主三副多节点”新格局成效显现，交通、水利、能源、市政、通信等基础设施更加完善，公路密度每百平方公里达126公里，信息网络、生态保障、节能减排等全面进步，新型城市化步伐明显加快。

　　——民生福祉实现新提升。人民生活更加殷实，城镇居民人均可支配收入年均增长8%，农村居民人均纯收入年均增长9%，城乡收入比进一步缩小，城镇化率接近90%，发展成果、公共服务、平等待遇“三个共享”水平明显提高。

　　——文化引领迈开新步伐。孙中山文化时代价值不断提升，全民修身行动成效明显，城乡一体公共文化服务体系健全，文化事业繁荣发展，文化产业增加值占生产总值比重达8.5%，城市文明水平整体提高，文化强市建设取得新进展。

　　——民主法治达到新水平。政府管理服务转型加快推进，政府架构更加优化、管理更加透明、决策更加科学、监督更加健全，城乡基层管理体制加快完善，社会自治水平逐步提高，公共安全得到有效保障，形成社会和谐人人有责、人人共享的生动局面。

　　2012年是新一届政府工作起始之年，是完成《珠三角规划纲要》“四年大发展”任务的决胜之年，做好今年工作意义重大。我们要全面贯彻落实中央经济工作会议、省委省政府工作部署和市第十三次党代会精神，坚持科学发展主题，把握加快转变经济发展方式主线，以率先加快转型升级、建设幸福和美中山为核心，围绕“稳增长、抓转型、强基础、优环境、促和谐、惠民生”工作重点，努力实现稳中求进，做到“稳”有保障、“进”有更大成效，促进经济平稳健康可持续增长，促进发展质量不断提高，促进人民群众幸福感不断增强。

　　2012年经济社会发展的主要预期目标是：生产总值增长11%，单位生产总值能耗下降3.9%，全社会固定资产投资增长16%，社会消费品零售总额增长16%，外贸出口总值增长7%，地方财政一般预算收入增长12%，城镇居民人均可支配收入增长8%，农村居民人均纯收入增长9%，居民消费价格涨幅在4%左右，城镇登记失业率控制在3%以内，人口自然增长率控制在7‰以下，节能减排约束性指标完成省下达年度计划。

　　为实现上述目标，必须做好以下九方面工作。

　　一、保持经济平稳健康可持续发展

　　提高发展质量效益。坚持“能耗低、占地少、用工精、创新多、效益高、生态好、惠民实”发展导向，市财政扶持产业发展资金增加到5亿元。发展实体经济，增强发展包容性。力争人均生产总值增长10%，高新技术产品产值占规模以上工业总产值比重达40%，全员劳动生产率、单位建设用地增加值等质量效益指标明显提升。科学统筹项目安排和用地指标、土地储备、环境容量、电力供应、扶持资金等生产要素。实施节约集约用地，新增用地指标与土地利用成效挂钩，盘活闲置用地。“三旧”改造年内实施1万亩，完成历史用地手续确认报批。

　　深入实施“三个一百”战略。加快外资及来料加工百强企业转型升级、内资百强企业做强做大、引进百强企业落地投产。加强招商引资，促进“3•28”和央企、民企、世界500强签约项目落地。落实发展总部经济系列政策，支持本土企业和吸引市外企业在中山建立总部。鼓励优势企业兼并重组，发展成长型科技型中小企业。做强企业做大品牌，从名牌名标培育行业冠军，打造更多十亿级、百亿级企业。工业增加值增长12%。

　　积极发展经济协作区。设立市级经济协作区试点，按照“市级统筹、镇区联动、利益均衡、整体发展”原则，完善税收增量共享等利益分配机制，打造承载优质项目新平台、提升发展质量新高地、专业镇升级新阵地，推动行政区经济向经济区经济发展。加强产业园区优化整合，做大华南现代中医药城、国家健康医药产业基地等优质园区。推进产业和劳动力“双转移”。

　　扩大投资促进消费。加大投资力度，优化投资结构。安排市重点项目126项，年度计划投资210.2亿元。扶持拥有自主知识产权与品牌的企业加大投资，争取上级财政、信贷资金支持，鼓励扩大民间投资。支持企业开拓内销市场，开展“中山产品全国行”，探索建设省外经贸合作平台，提高专业会展综合效益。增强消费拉动增长作用，发展绿色、健康、网络等新型消费。加快城轨站场商业开发，推进兴中广场、富华、益华等商圈建设。落实“菜篮子”工程，加快平价商店等“三项建设”。推动房地产市场健康稳定发展。维护市场价格秩序，加强社会信用建设，营造便利放心的消费环境。

　　稳定出口鼓励进口。加大出口市场开拓深度，扭转对欧美贸易下滑势头，开发香港、欧盟细分市场，拓展东盟、中东、非洲、拉美、俄罗斯等新兴市场，建设外贸企业信用融资平台，健全出口信用风险保障机制。完善促进进口政策，引导企业重点扩大省鼓励目录的技术和产品进口。优化口岸通关环境，推进保税物流中心申报综合保税区，做好国际贸易壁垒预警和应对工作。支持外贸企业转型升级，落实8个系列30项扶持政策，提升中山产品国际竞争力。

　　二、加快产业转型升级

　　改造提升传统优势产业。深入实施传统产业振兴、专业镇升级、产业集群壮大计划，推动灯饰照明、家用电器、五金锁具、休闲服装、红木家具等产业向高附加值环节延伸。建设好广东灯饰国际采购中心、小榄工业设计产业基地等省产业升级示范点。扶持若干产业集群建立价格指数，抢占行业定价权。运用信息化技术改造传统产业，建设黄圃小家电电子商务平台，开展企业精细化管理物联网技术应用试点。建设质量强市，实施市技术标准战略，鼓励企业参与制定国际标准和主导制定国家标准。

　　大力发展先进制造业。推动制造业高端化发展，重点培育电子信息、船舶制造和海洋工程、电梯、大型铸锻件、专用机械等制造业。加快中船中山基地、中铁南方工程装备、蒂森电梯、立信纺织染整机械等15个重点项目建设。力争临海装备制造业基地年内产值超百亿元，先进制造业增加值占规模以上工业增加值比重达33.9%。

　　培育发展战略性新兴产业。推动新兴产业规模化发展，重点培育新能源、新光源、高端信息技术、健康医药等产业。加快建设大型风力发电机组产业化基地、新能源汽车驱动系统产业化、大功率LED封装等高新技术产业重点项目。实施新能源汽车、半导体照明、智能电网、太阳能光伏等应用示范工程，建设绿色照明示范城市。促进国家软件服务示范基地、“北斗”卫星导航产业化基地落地。

　　加快发展现代服务业。推动服务业集聚化发展，拓宽服务业发展空间，加快重点集聚区、重点项目、重点企业建设，加强服务业统计。重点培育地方金融、信息服务、工业设计、现代物流、商务会展等业态，促进商贸餐饮、社区服务等品牌化规模化经营。加快建设广东省健康医疗信息技术服务区、华南家电产业服务创新区、中山国际金融中心、小榄金融大厦、灯饰产业综合服务区、世宇动漫产业园、苏宁物流等重点项目。提高东区中心商务区、西区服务业综合改革试验区、石岐综合试验区、广东软件和数字家庭孵化产业基地等集聚区辐射能力。服务业增加值增长12%，占生产总值比重达43%。

　　积极发展旅游业。加快翠亨国际旅游小镇建设，打造具有岭南特色、民国风情的国际旅游名镇。启动岐江游二期建设，丰富岐江游内涵。建设盛世游艇会、温泉宾馆改造等一批重点项目。办好岭南水乡旅游文化节、小榄菊花节、石岐休闲旅游文化节等活动，推广工业旅游、香山美食、体育保健、游艇休闲等特色旅游。打造区域自驾游基地。

　　扎实发展现代农业。市财政安排现代农业资金3.1亿元，发展节约型、效益型、生态型农业。促进农业与现代物流、电子商务、观光旅游融合，提升现代农业示范区内涵。与外市合作建立农产品基地。提高农业基础设施标准和农机化水平，增强农业抗灾能力和产出效益。建设“硬底化”主干农路195公里和高标准农田4000亩，完成危桥改造40座。严格农产品质量管理，强化动植物疫病防控。

　　三、提高城市化发展水平

　　努力建设理想城市。树立绿色发展、智慧发展、包容发展和人本发展理念，走文明、宜居、承载力和可持续发展能力强的新型城市化道路。按照城市定位精准、立面精致、管理精细原则，制定城市化发展工作方案，建设一批城市新区、宜居社区、文化街区、绿色建筑。实施母亲河工程，推进两岸城市综合功能开发，打造岐江流域“亲水、宜居、活力”长廊。加快智慧城市建设，推动空间地理系统应用，建设政务信息化云计算平台，完善医疗卫生、智能交通、数字城管等信息化系统。创新城市管理体制，提高城市科学化精细化管理水平。

　　加快翠亨新区建设。坚持科学开发、从容建设，以城市化带动工业化，以垂直整合促进产业组团升级，以智慧用海拓宽城市发展空间。编制新区总体规划和环境保护、交通防灾、地下空间利用等专项规划。理顺管理架构，确定开发模式，加快围填海步伐，推进一批重点项目，争取成为凭海域使用权证办理建设手续试点。建设海内外华人共有精神家园探索区、两岸四地更紧密合作示范区、新一代珠西岸理想城市先行区、智慧用海试验区，打造带动全市升级发展的新引擎。

　　强化科学规划引导。加强规划统筹，健全城乡规划体系，推动城市总体规划、土地利用规划、国民经济与社会发展规划“三规融合”。提高规划前瞻性、科学性、约束性，促进城市建设高起点、高效率、高品位。建立城市立面审批制度，培育特色鲜明城市风貌。出台主体功能区规划，按照“优化提升区、功能拓展区、协调发展区、生态保育区”格局，调整空间功能布局。提高主城区首位度，增强主城区集聚辐射作用，推进与主城区互动发展的东部、西北部、南部三大片区副中心建设。

　　构建综合交通运输体系。大力促进交通外拓、内联、畅行，落实“3015”工程，做好市际镇际道路对接，加快实现镇区30分钟到城区、15分钟上高速。配合推进深中通道、西部沿海铁路、中莞城际轨道、南沙铁路等规划建设，启动建设江番高速番禺大桥，开工建设古神公路二期、中山港大桥扩建、横二线东段、纵四线、纵二线南段等项目，加快建设广珠中线二期、105国道中山段改造、东部快线、小榄快线、新十水线古镇至南头段、南区中环路、北外环路改造等项目，广珠西线三期、福源路三角至港口段等年内投入使用。实施公交战略规划，加强交通组织管理，构建公交快速环线和换乘体系，建设主城区公交换乘中心，增加公交车投放。加快慢行系统规划建设。优化“一港五区”功能，建设黄圃港，实现中山港对外轮开放。

　　加强基础设施建设。强化能源保障，加快建设110千伏及以上输变电工程、镇区天然气利用工程、嘉明电厂三期、国电热电联产联供、黄圃热电联产扩建等项目。完善农村基础设施，加快建设张家边泵站、福隆泵站、三乡排涝工程、中珠联围海堤达标加固等项目。加强民生工程建设，推进北京积水潭中山骨科医院、市第二和第三人民医院扩建、市心理卫生中心、桂山中学重建、广东理工学院中山校区二三期等项目建设。

　　四、增强创新驱动能力

　　积极开展金融创新。推动金融资本、土地资本、产业资本“三资融合”，为吸引创新资源探索新路。加快城乡金融服务一体化综合改革试点建设，构建资本充足、功能完善、风险可控的金融服务体系。完成农村信用社改制为农村商业银行工作，发展村镇银行、小额贷款公司、融资性担保公司等本土金融机构，提高中小微企业和农户贷款比例。支持农业风险互助协会工作，扩大政策性农业保险覆盖面。实施企业上市梯队培育工程，支持上市公司再融资，争取火炬区进入“新三板”试点范围。打造珠江口西岸股权投资基金集聚区，引导社会资本进入实体经济。

　　推动“政产学研”协同创新。加大科技投入，研发经费占生产总值比重达2.2%。把市生产力促进中心、小榄生产力促进中心、火炬区高技术创业中心、市家电创新中心等发展成为区域科技服务平台。建立自主创新产品优先采购制度，激发企业创新动力。推动国家重点高校、科研院所在中山建设研发机构，重点提升北京理工、武汉大学、武汉理工、南京林大等研发平台服务能力。开展核心技术攻关，实施重大科技专项，推动科技成果产业化。加强知识产权保护，完善国家知识产权快速维权中心功能。加快发展民营科技园。

　　加快实施人才发展规划纲要。推进扶持创新团队、引进高端人才、开发技能人才等十大工程，落实引进培育紧缺适用人才“1+15”政策。建设全市人才信息库，做好省市共建留学人员创业园工作，鼓励设立国家重点实验室和院士工作站等人才载体。落实人才入户等优惠政策，提高人才服务水平。造就规模大、结构优、素质高的人才队伍，建设人才强市。

　　五、加强生态文明建设

　　实施绿化美化工程。加快主城区和镇区中心景观改造，形成具有岭南特色和水乡风格的生态景观。加强城镇出入口、主干道、主河道两侧和重要景区风貌综合整治，推进紫马岭东湖、田心森林、西区湿地等公园建设。继续开展“大种树、多种树、种好树”活动，实施生态景观林带示范段工程。完善林权制度改革，完成6500亩林分改造和23条村“万村绿”建设，人均公园绿地面积达10.5平方米。完善主城区绿道网配套设施，启动镇区绿道建设。

　　实施碧水蓝天工程。全面加快治水工程，启动水利现代化试点建设。综合整治内河涌200公里，改善岐江河水质。实施主城区雨污分流工程，提高生活污水设施运营效能，推进珍家山、中嘉污水收集系统二期建设。强化水资源安全，严格落实饮用水源地保护措施，探索分质供水。加大治咸力度，推进南部地区抗咸保供水工作。整治重金属和畜禽养殖污染。加强大气污染防治，做好机动车尾气和微尘排放监管。

　　实施节能减排工程。优化能源结构，淘汰落后产能，发展环保产业，落实固定资产投资项目节能评估审查制度。推行清洁生产，引进和创新低能耗低排放生产技术。严格用能管理，实施节能减排重点工程，抓好建筑、交通、商贸、公共机构和农村等非工领域节能。建设小榄低碳发展促进中心、城区汽车天然气加气站，倡导绿色消费和节能生活。

　　实施秀美村庄工程。加快农村环境综合整治、生活设施配套、乡村景观改造、田园风光维护和绿化美化建设，落实村庄规划，推动农村社区化管理。加快建设名镇名村，按照“生活富裕、环境优美、乡风文明、特色鲜明”原则，做好4镇6村（社区）试点工作，扩大镇村示范面。

　　六、创新社会管理工作

　　构建多元共治新格局。完善“一个理念、两个突破、三个共享”社会管理模式。统筹全市社会管理，构建“党委统一领导、政府主导推动、民间组织运作、公众广泛参与”的社会工作发展格局，增加外来务工人员平等参与社会管理机会，推动社会管理和谐善治。

　　培育发展社会组织。社会组织业务主管单位逐步转为业务指导单位，允许社会组织直接向民政部门申请成立。创建社会组织发展专项基金和孵化基地，通过资金扶持、转移职能、购买服务等方式，支持发展行业协会、农村专业经济协会、社区社会组织和公益慈善团体。积极开展社会组织等级评估，推进社会组织规范化发展。

　　提升社区建设水平。建设镇区政务服务中心和社区（村）政务服务代办点。开展和谐企业、和谐家庭、和谐社区创建活动，形成乐工作、亲邻里、优生活的社区新貌，加大“六好”平安和谐社区创建力度。推广社区（村）管理“2＋8＋N”模式，推进社区事务一站式服务。理顺社区管理与物业管理关系。

　　加强社工队伍建设。发展社会工作机构，加强组织架构、服务质量、绩效评估等标准化建设。构建本土化专业化社工队伍，引进培养社工高端人才，完善社工专业培训体系，鼓励本地高校开设社会工作专业。发展志愿者组织，推行“社工＋义工”模式，建立社工引领志愿者服务体系。

　　建设和谐平安中山。健全适应社会转型的权益保障机制，完善人民调解、劳动调解、行政调解、司法调解互相衔接的“大调解”格局。依法化解社会矛盾，做好新形势下群众工作，关注新生代农民工问题，构建和谐社会关系。做好来信来访工作，加强危机应急管理，强化虚拟社会管理。开展心理健康服务，培养理性、平和、包容的社会心态。加强社会治安综合整治，严厉打击违法犯罪。落实安全生产责任，遏制重特大事故，加强气象灾害等突发事件预警，确保人民群众生命财产安全。

　　七、推进文化强市建设

　　弘扬孙中山文化。凝聚华侨华人力量建设伟人故里。创新文化“走出去”模式，把孙中山文化节、“中山杯”华侨华人文学奖、翠亨论坛等活动办成交流合作平台，提升中山文化实力与影响力，努力打造建设文化强省的示范市、展现岭南文化的精品市、构建共有精神家园的先行市。

　　深入开展全民修身行动。培养文化自信，形成文化自觉，激励文化自强。用社会主义核心价值体系引领社会思潮，推进公民意识培育、城市精神光大、优良品德倡导、公共文明实践、传统文化弘扬、幸福能力提升、新老中山人融合、阳光少年自强、领导干部尚德、和美环境营造“十大行动”，制定《全民修身行动测评体系》。以全国文明城市复检为契机，全面增强城市软实力。

　　加大公共文化普惠力度。动工建设139文化街区、漫画馆、中山纪念图书馆，加快建设非物质文化遗产展览馆二期、城市雕塑。健全市镇村三级公共文化服务网络，力争每万人拥有公共文化设施面积1200平方米。建设镇区现代新型综合文化站，提升改造农村文化室。优化城市“十分钟文化圈”和农村“十公里文化圈”，加强文物保护单位和重要历史建筑的保护利用。

　　繁荣文化事业。做好文化体制改革工作。积极创作体现时代精神和本土特色的文学艺术精品。擦亮中国合唱基地、城市论坛、社区文化艺术节、中山书展等一批有影响力的地方文化品牌，广泛开展群众文化活动，支持文化企业、社会力量提供优质公共文化产品。提升灯饰、家电、红木家具、休闲服装等特色产业文化内涵，扶持创意设计、教育培训、文化旅游、动漫游艺等文化产业做大做强。

　　八、提高民生福祉水平

　　完善社会保障体系。市财政安排5200万元促进就业谋福和创业富民，年内城镇新增就业5万人。提高中等收入者比重，保障低收入群体基本生活。稳步增加公益事业岗位，完善临时补贴、最低生活保障、失业保险标准与物价上涨挂钩联动机制，健全职工工资集体协商、正常增长和支付保障机制。加强劳务合作，保障企业用工需求。市财政投入3.9亿元用于社会保险，完善医疗保险、生育保险、工伤保险制度，探索引入商业保险、企业化管理参与医保服务。构建商品房和保障房双轨并行的新型住房制度，逐步解决低收入群众、新就业职工、外来员工住房困难，建立农村困难家庭住房改造长效机制。市财政安排9700万元用于社会救助，做好国家养老服务业标准化试点工作。加强红十字会和慈善总会建设，发展残疾人事业。

　　促进教育均衡优质发展。实施学前教育惠民扩优、义务教育均衡优质、普通高中优质集群发展、职业教育创优增效、高等教育内涵拓展、终身教育普惠共享、特殊教育提升发展、强师兴教、数字化教育等九大工程，增加优质教育资源供给，加快教育现代化。落实学前教育三年行动计划，发展惠普性公益性幼儿园。深化教育体制改革，打造广东省职业技术教育综合改革试验区，完善分级办学分级管理、生均经费市镇共担等模式，探索市级统筹高中阶段教育。扶持民办学校优质发展。

　　加强医疗卫生和食品安全工作。深化医疗卫生体制改革，全面实施国家基本药物制度，推进公立医院和基层医疗卫生机构综合改革。完善公共卫生预防保健服务体系，落实公共卫生服务项目及重大疾病防控、国家免疫规划、卫生应急等工作。鼓励社会资本举办医疗卫生机构，规范民营医疗机构管理。加大食品药品安全治理力度，强化对问题多发场所和大宗食品监管，坚决遏制危害人民群众健康的重大食品安全问题发生。

　　大力发展社会事业。制定人口均衡发展管理办法，科学调控人口规模，提高人口质量，开展流动人口计划生育基本公共服务均等化试点。创建全国全民健身示范城市，发展群众体育运动，提升竞技体育水平。做好“双到”扶贫开发和对口援建工作。充分发挥外事侨务、港澳事务、台务、民族宗教工作服务经济社会发展的重要作用，做好国防建设、双拥、妇女儿童、老龄、人防、气象、地震、档案、方志等工作。

　　九、深化重点领域改革

　　全力完成“四年大发展”目标任务。贯彻《珠三角规划纲要》，狠抓67个重点项目建设和39项指标落实，力争考核取得优异成绩。在环珠江口区域合作中争取主动，积极承接港澳穗深辐射，着力引进港澳高端服务业。做好港珠澳大桥对接工作，参与创建大珠三角优质生活圈，深化珠中江经济圈合作。

　　完善经济运行调节机制。加强经济运行态势研判，建立全市经济运行监测系统。发挥统计审计作用，提升预测预警水平。推动涉税信息共享，强化预算监控调节，提高财政支出绩效，完善镇区财政管理指导机制。鼓励民间资本参与设立村镇银行、小额贷款公司、融资性担保公司等金融机构。加大市属国有资产整合力度。推行政府投资项目代建制。

　　深化行政管理体制改革。优化组织结构，理顺职责关系，提高整体效能。落实简政强镇事权改革，开展第二批事权下放，建立动态扩权长效机制。分步推进审批事项延伸到镇区、社区（村）办理，逐步向社会组织转移服务事项。健全行政审批监督和绩效考核制度，拓展电子监察领域。

　　推进城乡一体化改革。提升城乡规划建设、基础设施、产业布局、公共服务、社会管理“五个一体化”水平，加快火炬区、西区、小榄、南头、沙溪、三乡6个镇区示范点建设。加强农村土地承包经营权流转管理，规范推进集体建设用地使用权交易。实施城乡户籍登记制度改革，缩小农村居民与城市居民福利待遇差距。推动流动人口积分服务向医疗社保、住房保障延伸，稳步提高外来务工人员子女免费入读义务教育公办学校比例，破解城乡双重二元结构取得新进步。

进一步提高政府自身建设水平

　　坚持勤政为民，建设服务政府。践行全心全意为人民服务宗旨，倾听群众呼声，关心群众疾苦，解决好涉及人民群众切身利益的问题。进一步转变政府职能，提高公共服务质量。坚持各级领导联系重点项目和企业制度，营造“亲商安商、扶商富商”浓厚氛围，帮助企业克服困难、加快发展。

　　明确改革导向，建设效率政府。采取措施多管齐下，克服互相推诿、办事拖拉和文山会海等问题，提高政府办事效率。坚持用效能意识改革公共部门，运用预算约束、绩效评估等手段，促使公共财政使用效益最大化。出台措施严控政府编制及人员增长，努力降低行政成本。

　　加强作风建设，建设阳光政府。保持真抓实干、狠抓落实的作风，增强敢于担当、善谋会干的本领，建设修身立德、团结奋进的队伍，全面提高政府执行力。积极推进政务公开和网络问政，保障群众知情权、参与权、表达权和监督权。

　　严格依法行政，建设法治政府。自觉接受人大依法监督和政协民主监督，提高议案建议提案办理质量。认真听取各民主党派、工商联、无党派人士和各人民团体意见，健全公众参与、专家论证、社会公示、风险评估、合法性审查和跟踪反馈等决策机制。落实行政处罚自由裁量权量化标准，深化行政复议委员会机制建设。实施“六五”普法，加强民主法治建设。

　　强化反腐倡廉，建设廉洁政府。完善惩治和预防腐败体系，探索建立廉政风险防控机制。强化行政监察，加强审计监督，实施重要项目资金使用常态监督，开展人财权物等资源密集重点领域专项治理，坚决纠正损害群众利益的不正之风。厉行勤俭节约，严控行政费用支出。

　　坚持把发展成果转化为民生福祉。我们去年全面实现36项内容的十件民生实事年度目标，提高公交出行分担率、改善岐江河水质、开通岐江游、开展全民植树、建设绿道网和慢行系统等成效深入人心。在此，我代表市人民政府承诺，在巩固提升去年阶段性成果基础上，今年继续办好十件民生实事。一是加强综合治水。启动全市雨污分流工作，在主城区开展安栏路污水主管等12项工程建设，建成管径600毫米以上污水管25公里。加快岐江河滨水景观整治二期工程建设，启动翠亨兰溪河改造。二是推进绿色出行。年内完成主城区58.5公里绿道建设，新投放公共自行车3000辆、新能源公共汽车100辆，实施公交卡乘车优惠。三是维护食品安全。加强绿色食品无公害产品认定，启动大宗食品安全检测体系建设，推动食品安全监督进社区。四是落实安居保障。筹建各类保障性住房4500套，完成405户低保低收入家庭危房改造。五是建设平安校园。全面完成中小学校舍安全工程。强化校园周边治安综合治理。加强校车安全，全市校车100%安装监控摄像头和卫星定位行驶记录仪。六是丰富基层文化。广泛开展文化下乡活动，建设5个市图书馆镇区分馆、10个示范农村文化室、10个示范文化广场、10个基层示范文艺社团、10个示范农家书屋。七是推行全民健身。市镇两级公共体育场馆免费或优惠开放，逐步扩大免费国民体质监测范围，开展经常性群众体育竞赛。八是实施稳价惠民。推动平价商店进镇区，增加平价商店40家，提高低收入群体补贴水平。九是做好养老助残。100%保障城镇“三无”人员基本生活，新增老年人应急呼叫装置3万个，增设残疾人、未成年人、单亲家庭等救助项目，提高城乡低保标准。十是完善治安防控。启动社会治安视频监控系统三期工程，新建整合一类监控点900个，减少社会面治安问题发生。
